# Impacts of Betaine Addition in Sow and Piglet's Diets on Growth Performance, Plasma Hormone, and Lipid Metabolism of Bama Mini-Pigs

**DOI:** 10.3389/fnut.2021.779171

**Published:** 2021-12-23

**Authors:** Yating Cheng, Mingtong Song, Qian Zhu, Md. Abul Kalam Azad, Qiankun Gao, Xiangfeng Kong

**Affiliations:** ^1^Hunan Provincial Key Laboratory of Animal Nutritional Physiology and Metabolic Process, Institute of Subtropical Agriculture, Chinese Academy of Sciences, Changsha, China; ^2^University of Chinese Academy of Sciences, Beijing, China; ^3^Research Center of Mini-Pig, Huanjiang Observation and Research Station for Karst Ecosystems, Chinese Academy of Sciences, Huanjiang, China

**Keywords:** Bama mini-pigs, betaine, growth performance, lipid metabolism, plasma hormone

## Abstract

The present study evaluated the effects of betaine addition in sow and piglet's diets on growth performance, plasma hormone, and lipid metabolism of Bama mini-pigs. A total of 26 pregnant Bama mini-pigs and 104 weaned piglets were selected and divided into different dietary treatment groups (details in “Materials and Methods”). Blood and muscle samples were collected at 65-, 95-, and 125-day-old, respectively. The results showed that betaine addition in sow-offspring diets increased (*P* < 0.05) the body weight at 125-day-old, average daily gain from 35- to 65-day-old, and average daily feed intake at 35–65 and 35–95 days old of pigs compared with the control group. Betaine addition in sow-offspring diets increased (*P* < 0.05) the plasma gastrin level at 95-day-old, while betaine addition in sow diets decreased (*P* < 0.05) the plasma peptide YY and leptin levels at 65-day-old pigs. In the *longissimus dorsi* muscle of pigs, betaine addition in sow and sow-offspring diets increased (*P* < 0.05) the C12:0 content at 65-day-old while decreased at 95-day-old. Moreover, betaine addition in sow-offspring diets increased the C24:0 content and decreased the C18:1n9t content at 125-day-old (*P* < 0.05). In the *biceps femoris* muscle, the contents of C12:0 at 65-day-old and C20:4n6 at 125-day-old were decreased (*P* < 0.05) after the betaine addition in both sow and piglet's diets. In addition, betaine addition in sow diets decreased (*P* < 0.05) the C20:0 content at 125-day-old, while betaine addition in sow-offspring diets increased the C18:3n6 and decreased C24:0 contents at 65-day-old pigs (*P* < 0.05). In the *psoas major* muscle, betaine addition in sow and sow-offspring diets decreased (*P* < 0.05) the contents of C18:1n9t at 65-day-old and C20:1 at 95-day-old, while betaine addition in sow diets decreased (*P* < 0.05) the intramuscular fat content at 125-day-old. Moreover, betaine addition in sow-offspring diets was also associated with muscle lipid deposition and metabolisms by regulating the gene expressions related to fatty acid metabolism. These findings suggested that betaine addition in sow-offspring diets could improve the growth performance, whereas betaine addition in both sow and sow-offspring diets could enhance lipid quality by altering plasma hormone level and fatty acid composition and regulating the gene expressions related to fatty acid metabolism.

## Introduction

Intramuscular fat (IMF) plays an important role in various aspects of meat quality, and it is also critical for the nutritional value of meat (1). Higher content of IMF can increase the meat quality as it contributes to pork tenderness, flavor, and juiciness (2). Furthermore, muscle fatty acid profiles also play a key role in meat quality because of their high value for human health. Polyunsaturated fatty acids (PUFA), especially n-3 fatty acids, are considered as functional ingredients to prevent cardiovascular disease in humans (3). The n-3 PUFA deficiency and excessive content of n-6 PUFA are associated with the development of insulin resistance and metabolic disorders (4). Therefore, abnormal lipid metabolism could inhibit the growth and decrease the meat quality of animals. In addition, a recent study has reported that changes in fatty acid composition due to different feeding strategies are associated with the changes in the mRNA expression of genes related to fatty acid metabolism (5). For example, fatty acid synthase (*FAS*) and stearoyl coenzyme A desaturase (*SCD*) genes are associated with the regulation of fatty acid biosynthesis, while lipoprotein lipase (*LPL*) can regulate the fatty acid transportation from blood to fat cell (6, 7). Thus, the regulation of IMF content and fatty acid composition in muscles has been of great interest in recent years. Furthermore, antibiotics are commonly used in the feed industry to promote the growth of animals. Owing to the ban on in-feed antibiotics in the livestock industry, a number of alternative feed additives with similar effects to in-feed antibiotics in livestock production have attracted increased attention.

Betaine is a common term for trimethylglycine, a substrate for betaine-homocysteine methyltransferase (HMT) in liver and kidney (8). As a promising antioxidant agent, betaine can prevent lipid peroxidation and regulate lipid metabolism to improve growth performance and meat quality. Gheisari et al. (9) reported that the betaine addition (1 g/kg) to the methionine-deficient diet could significantly improve antioxidant defense and meat quality by decreasing lipid peroxidation in breast muscles of broilers. In addition, dietary betaine could improve the meat quality of lambs, and rumen-protected betaine exhibited better effects than that of the unprotected-betaine (10). However, there are limited studies about maternal betaine addition on offspring's growth performance and lipid metabolism.

Bama mini-pig is a high-quality local pig breed in China because of its characteristics of delicious meat, high roughage tolerance, and good adaptability. However, the lower dietary nutrient level and extensive management methods have resulted in lower growth, feed conversion, and lean meat rate of this mini-pig. Our previous studies showed that 3.50 kg/t betaine addition to sow and sow-offspring diets could increase the carcass weight, carcass yield, lean meat rate, meat color, shear force, and crude protein content of muscles at different stages by altering the plasma biochemical parameters and amino acid composition, and regulating the expression level of genes related to myosin heavy-chain (MyHC) isoform and myogenic regulatory factors (MRFs) in skeletal muscle (11). Moreover, dietary betaine hydrochloride addition (3.50 kg/t) to sows from gestation to lactation could improve suckling piglet's health by enhancing intestinal morphology and immunity and altering intestinal microbiota (12). Therefore, we hypothesized that dietary betaine might influence the growth performance and meat quality of pigs by regulating the lipid content, IMF content, and mRNA expression of genes related to fatty acid metabolism in skeletal muscle. Thus, the current study was conducted to test our hypothesis that the addition of dietary betaine to sow and sow-offspring would improve the offspring's growth performance and thereby influence the lipid metabolism. Furthermore, to explain the mechanism of dietary betaine addition on growth performance and lipid metabolism of Bama mini-pigs by evaluating plasma hormones and the fatty acid metabolism-related genes.

## Materials and Methods

### Animals, Diets, and Experimental Design

Twenty-six pregnant Bama mini-pigs with similar body weight (BW) were selected and randomly divided into control group (sows fed a basal diet; *n* = 12) and betaine group (sows fed a basal diet supplemented with 3.50 kg/t betaine hydrochloride; *n* = 14). The sows were housed individually in gestation crates (2.2 × 0.6 m) from mating to day 104 of gestation. On day 105 of gestation, the sows were transferred to individual farrowing crates (2.2 × 1.8 m) with a heated floor pad for offspring piglets with freely accessible water. After weaning, at 35-day-old, a total of 104 piglets with an average BW of litters were selected from the two groups (48 piglets from the control group and 56 piglets from the betaine group) and divided into three dietary treatments as follows: a) control group, piglets from the control group fed a basal diet (*n* = 48); b) sow betaine group, piglets from the betaine group fed a basal diet (*n* = 28); and c) sow-offspring betaine group, piglets from the betaine group fed a basal diet with 2.50 kg/t betaine hydrochloride (*n* = 28). Four piglets were housed in one pen, and the rearing compartments were temperature-controlled (23–26°C) and had forced ventilation. Betaine hydrochloride (purity ≥ 95%) was purchased from Sunwin Biotech Shandong Co., Ltd (Shandong, China). The betaine was mixed with the basal diets before feeding the sows and piglets. The supplementing dose of betaine was determined considering the tolerance of sows and piglets and based on previous studies (13, 14).

During the trial, the sows were fed pregnant diets from day 3 after mating to day 104 of pregnancy and fed lactating diets from day 105 of pregnancy to weaning. The piglets were fed pre-nursery diets from 35- to 95-day-old and fed late-nursery diets from 96- to 125-day-old. The nutrient levels of basal diets for sows and piglets met the Chinese local swine nutrient requirements (NY/T 65-2004), and the premixes met the National Research Council (NRC, 2012) diet requirements (15, 16). The composition and nutrient levels of basal diets for sows and piglets are presented in Supplementary Tables 1, 2, respectively. The sows were fed twice daily (at 08:00 and 17:00) and changed with their body condition. The piglets were fed four times daily (at 08:00, 12:00, 15:00, and 19:00), and the dietary stage was based on the physiological stage of piglets. All animals had *ad libitum* access to drinking water throughout the trial.

### Sample Collection

The animals were weighed at 65-, 95-, and 125-day-old after a 12 h fasting, respectively. A total of 26 pigs (including 12, 7, and 7 pigs from the control group, sow betaine group, and sow-offspring betaine group, respectively) were selected at each stage (65-, 95-, and 125-day-old) and sacrificed under commercial conditions *via* electrical stunning (120 V, 200 Hz) and exsanguination (17). Blood samples (5 mL) were collected from the anterior vena cava, anticoagulated with heparin, and centrifuged at 2 000 × *g* for 10 min to obtain plasma. After slaughter, *longissimus dorsi* (LD), *biceps femoris* (BF), and *psoas major* (PM) muscles were sampled. One part of the muscle samples was stored at −20°C for measuring IMF content and fatty acid composition, and another part was stored at −80°C for analyzing the expression level of genes related to fatty acid metabolism.

### Growth Performance

During the trial, the feed intake of each pen was recorded daily, and the BW (12 h fasting) was taken at 65-, 95-, and 125-day-old. The average daily gain (ADG), average daily feed intake (ADFI), and feed/gain ratio (F:G) were calculated.

### Plasma Hormone Analysis

The levels of plasma hormones, including cholecystokinin (CCK), gastrin (Gas), growth hormone (GH), insulin-like growth factor (IGF), leptin (LEP), pancreatic polypeptide (PP), peptide YY (PYY), and somatostatin (SS) were measured using commercially available enzyme-linked immunosorbent assay kits (Jiangsu Yutong Biotechnology Co., Ltd., Jiangsu, China) on a Multiscan Spectrum Spectrophotometer (Infinite M200 Pro; Tecan, Männedorf, Switzerland) in accordance with the manufacturers' instructions.

### Intramuscular Fat and Fatty Acid Analysis

The muscle samples were cut into thin slices, dried in a vacuum freeze dryer at (10 ± 5) Pa and –(45 ± 5)°C for 48 h, and then ground into powder. The IMF was measured according to GB/T 9695.7-2008 (technical manual for testing the total ether extract contents in the meat). The composition of medium-chain fatty acids (MCFA) and long-chain fatty acids (LCFA) in muscles were measured by using gas chromatography as previously described by Li et al. (18).

### Analysis of the Expression Levels of Genes Related to Fatty Acid Metabolism in Muscles

Total RNA was isolated from LD, BF, and PM muscles using TRIzol (Invitrogen, Shanghai, China). β-actin and target genes based on related cDNA sequences of pigs in the GeenBank database in NCBI are presented in Supplementary Table 3, and the gene-specific primers were synthesized by Invitrogen Biotech Co., Ltd. (Shanghai, China). The reverse transcription-polymerase chain reaction (RT-PCR) assays were conducted using the SYBR Premix Ex Taq™ Kit (TaKaRa Biotechnology Co., Ltd., Dalian, China). The RNA quality detection, including the RT-PCR and cDNA synthesis, was used the same methods as described in the previous study (19). The RT-PCR conditions were as follows: initial denaturation at 95°C for 30 s, followed by 40 cycles of denaturation at 95°C for 5 s and annealing at 60°C for 30 s, and finally extension at 72°C for 30 s. The relative gene expression was calculated using the 2^−ΔΔCT^ method (20).

### Statistical Analysis

The data analyses were performed with SPSS 22.0 software (IBM Corporation, Armonk, NY, USA) using a one-way analysis of variance (ANOVA). The means of different groups were compared by Duncan tests. All data are presented as means ± SE unless otherwise indicated. Differences were considered statistically significant when *P* < 0.05.

## Results

### Growth Performance

The growth performance of pigs is presented in Table 1. The BW in the sow-offspring betaine group was increased (*P* < 0.05) at 125-day-old compared with the control group and at 95-day-old compared with the sow betaine group. The ADG from 35- to 65-day-old and ADFI from 35- to 65- and from 35- to 95-day-old were increased (*P* < 0.05) in the sow-offspring betaine group compared with the other two groups. Moreover, the ADG was increased (*P* < 0.05) in the sow-offspring betaine group from 35- to 95-day-old compared with the sow betaine group. However, dietary betaine addition in sow and sow-offspring diets did not affect (*P* > 0.05) the F:G of piglets.

**Table 1:** Effect of dietary betaine addition on growth performance of Bama mini-pigs.

**Items**	**Day-old**	**Control group**	**Sow betaine group**	**Sow-offspring betaine group**
BW (kg)	35	4.94 ± 0.17	4.99 ± 0.36	4.70 ± 0.17
	65	9.11 ± 0.25	9.77 ± 0.31	9.72 ± 0.12
	95	14.64 ± 1.07^ab^	12.24 ± 0.80^b^	17.06 ± 1.07^a^
	125	22.66 ± 2.69^b^	27.00 ± 1.79^ab^	32.46 ± 2.55^a^
ADG (kg)	35–65	0.15 ± 0.01^b^	0.16 ± 0.01^b^	0.19 ± 0.01^a^
	35–95	0.16 ± 0.01^ab^	0.13 ± 0.01^b^	0.20 ± 0.02^a^
	35–125	0.21 ± 0.03	0.22 ± 0.02	0.29 ± 0.03
ADFI (kg)	35–65	0.41 ± 0.01^b^	0.39 ± 0.02^b^	0.47 ± 0.02^a^
	35–95	0.51 ± 0.02^b^	0.53 ± 0.04^b^	0.66 ± 0.02^a^
	35–125	0.66 ± 0.05	0.69 ± 0.05	0.82 ± 0.03
F:G	35–65	2.84 ± 0.13	2.51 ± 0.12	2.53 ± 0.16
	35–95	3.66 ± 0.34	4.28 ± 0.25	3.49 ± 0.31
	35–125	3.67 ± 0.61	3.10 ± 0.09	2.89 ± 0.25

*Data are presented as means ± SE. Values in the same row without a common superscript letter are different (P < 0.05). Control group, n = 12, 11, and 8; sow betaine group, n = 6, 7, and 6; sow-offspring betaine group, n = 7, 7, and 6; at 65-, 95-, and 125-day-old, respectively. ADG, average daily gain; ADFI, average daily feed intake; BW, body weight; F:G, feed/gain ratio*.

### Plasma Hormone Level

The plasma hormone levels of pigs at different stages are presented in Table 2. Betaine addition in sow-offspring diets increased (*P* < 0.05) the plasma Gas level of 95-day-old pigs compared with the other two groups. Compared with the control group, betaine addition in sow diets decreased (*P* < 0.05) the plasma PYY and LEP levels of 65-day-old pigs. There were no significant differences (*P* > 0.05) in the plasma hormone levels of CCK, GH, IGF, PP, and SS at different stages among the three groups.

**Table 2:** Effect of dietary betaine addition on plasma hormone levels of Bama mini-pigs.

**Items**	**Day-old**	**Control group**	**Sow betaine group**	**Sow-offspring betaine group**
CCK (pg/mL)	65	940.73 ± 83.74	627.35 ± 39.32	846.33 ± 53.10
	95	498.05 ± 25.09	503.02 ± 29.26	491.08 ± 16.63
	125	607.61 ± 71.89	630.29 ± 71.47	492.72 ± 47.82
Gas (ng/mL)	65	0.81 ± 0.08	0.60 ± 0.07	0.66 ± 0.04
	95	0.84 ± 0.06^b^	0.89 ± 0.08^b^	1.30 ± 0.15^a^
	125	1.04 ± 0.13	0.87 ± 0.03	0.82 ± 0.05
GH (ng/mL)	65	45.77 ± 3.21	33.60 ± 6.45	32.43 ± 3.86
	95	29.91 ± 2.26	27.36 ± 1.88	29.27 ± 2.24
	125	29.85 ± 2.69	33.49 ± 3.91	25.26 ± 0.87
IGF (ng/mL)	65	228.87 ± 16.06	167.31 ± 31.57	162.16 ± 19.30
	95	146.49 ± 10.90	151.52 ± 7.65	141.63 ± 6.59
	125	190.08 ± 21.78	189.96 ± 19.03	179.96 ± 12.59
LEP (ng/mL)	65	30.44 ± 2.08^a^	22.26 ± 0.83^b^	25.99 ± 2.22^ab^
	95	19.29 ± 1.06	20.68 ± 1.52	22.58 ± 2.05
	125	22.41 ± 2.16	19.45 ± 0.71	20.77 ± 1.56
PP (ng/mL)	65	4.14 ± 0.38	3.01 ± 0.55	2.96 ± 0.22
	95	2.99 ± 0.23	2.89 ± 0.16	3.00 ± 0.14
	125	2.94 ± 0.27	2.45 ± 0.11	2.44 ± 0.09
PYY (pmol/L)	65	20.37 ± 1.58^a^	14.54 ± 0.71^b^	16.62 ± 1.30^ab^
	95	15.74 ± 1.03	17.98 ± 1.54	18.14 ± 1.73
	125	18.21 ± 2.37	16.61 ± 0.90	17.07 ± 1.27
SS (pg/mL)	65	171.46 ± 14.73	136.07 ± 25.40	124.67 ± 9.12
	95	160.66 ± 15.48	154.64 ± 12.12	167.31 ± 14.43
	125	112.21 ± 6.60	163.88 ± 21.68	168.48 ± 32.71

*Data are presented as means ± SE. Values in the same row without a common superscript letter are different (P < 0.05). Control group, n = 11, 11, and 8; sow betaine group, n = 6, 7, and 6; sow-offspring betaine group, n = 7, 7, and 5; at 65-, 95-, and 125-day-old, respectively. CCK, cholecystokinin; Gas, gastrin; GH, growth hormone; IGF, insulin-like growth factor; LEP, leptin; PP, pancreatic polypeptide; PYY, peptide-YY; SS, somatostatin*.

### Intramuscular Fat Content

The IMF content in the LD, BF, and PM muscles is presented in Tables 3–5, respectively. The sow betaine group had a lower (*P* < 0.05) IMF content in the PM muscle of 125-day-old pigs compared with the other two groups. There was no significant difference in the IMF content in the LD and BF muscles among the three groups (*P* > 0.05).

**Table 3:** Effect of dietary betaine addition on medium- and long-chain fatty acid contents in *longissimus dorsi* muscle of Bama mini-pigs (fresh weight basis; %).

**Items**	**Control group**	**Sow betaine group**	**Sow-offspring betaine group**
**65-day-old**			
Intramuscular fat	2.55 ± 0.18	2.62 ± 0.26	2.67 ± 0.22
C12:0	0.04 ± 0.01^b^	0.05 ± 0.00^a^	0.05 ± 0.01^a^
C14:0	0.49 ± 0.10	0.45 ± 0.06	0.41 ± 0.04
C15:0	0.03 ± 0.01	0.02 ± 0.01	0.02 ± 0.02
C16:0	8.20 ± 0.79	7.58 ± 0.42	7.53 ± 0.35
C16:1	1.16 ± 0.16	1.10 ± 0.23	1.10 ± 0.26
C17:0	0.12 ± 0.07	0.12 ± 0.03	0.11 ± 0.02
C18:0	4.27 ± 0.36	4.04 ± 0.36	4.15 ± 0.39
C18:1n9c	8.87 ± 1.21	8.38 ± 0.51	8.28 ± 0.88
C18:1n9t	0.04 ± 0.01	0.04 ± 0.01	0.04 ± 0.01
C18:2n6c	4.17 ± 0.49	4.03 ± 0.50	4.48 ± 0.68
C18:3n3	0.17 ± 0.02	0.17 ± 0.02	0.16 ± 0.01
C18:3n6	0.02 ± 0.01^ab^	0.03 ± 0.02^a^	0.01 ± 0.02^b^
C20:0	0.06 ± 0.02	0.06 ± 0.01	0.05 ± 0.01
C20:1	0.24 ± 0.08	0.19 ± 0.02	0.19 ± 0.03
C20:2	0.16 ± 0.03	0.14 ± 0.01	0.15 ± 0.03
C20:3n6	0.10 ± 0.03	0.09 ± 0.01	0.12 ± 0.01
C20:4n6	0.81 ± 0.39	0.71 ± 0.09	1.06 ± 0.24
C22:6n3	0.04 ± 0.02	0.04 ± 0.01	0.02 ± 0.03
C24:0	0.05 ± 0.02	0.05 ± 0.03	0.07 ± 0.05
**95-day-old**			
Intramuscular fat	2.40 ± 0.31	2.83 ± 0.38	3.07 ± 0.27
C12:0	0.04 ± 0.01^a^	0.03 ± 0.01^b^	0.03 ± 0.01^b^
C14:0	0.49 ± 0.08	0.41 ± 0.19	0.47 ± 0.22
C15:0	0.02 ± 0.01	0.03 ± 0.01	0.02 ± 0.00
C16:0	8.26 ± 0.78	8.22 ± 0.56	8.49 ± 0.57
C16:1	0.71 ± 0.07	0.81 ± 0.13	0.76 ± 0.14
C17:0	0.10 ± 0.04	0.11 ± 0.03	0.09 ± 0.02
C18:0	5.13 ± 0.64	5.00 ± 0.29	5.08 ± 0.85
C18:1n9c	8.81 ± 1.10	8.91 ± 0.97	9.45 ± 1.47
C18:1n9t	0.03 ± 0.01	0.03 ± 0.01	0.03 ± 0.01
C18:2n6c	3.29 ± 0.86	3.29 ± 0.72	2.98 ± 0.45
C18:3n3	0.13 ± 0.01	0.13 ± 0.02	0.12 ± 0.02
C20:0	0.07 ± 0.02	0.07 ± 0.01	0.07 ± 0.01
C20:1	0.30 ± 0.08	0.25 ± 0.05	0.35 ± 0.09
C20:2	0.15 ± 0.02	0.13 ± 0.02	0.15 ± 0.02
C20:3n6	0.09 ± 0.04	0.09 ± 0.03	0.07 ± 0.02
C20:4n6	0.66 ± 0.53	0.60 ± 0.19	0.46 ± 0.11
C24:0	0.02 ± 0.03	0.03 ± 0.02	0.03 ± 0.02
**125-day-old**			
Intramuscular fat	2.48 ± 0.33	2.49 ± 0.49	2.57 ± 0.21
C12:0	0.03 ± 0.01	0.03 ± 0.01	0.02 ± 0.01
C14:0	0.51 ± 0.12	0.47 ± 0.10	0.44 ± 0.10
C16:0	8.22 ± 0.99	8.22 ± 1.25	7.54 ± 0.57
C16:1	0.82 ± 0.14	0.80 ± 0.15	0.76 ± 0.10
C17:0	0.09 ± 0.03	0.10 ± 0.05	0.07 ± 0.02
C18:0	4.73 ± 0.54	5.01 ± 1.23	4.44 ± 0.25
C18:1n9c	9.79 ± 1.00	10.01 ± 1.71	8.83 ± 0.58
C18:1n9t	0.043 ± 0.01^a^	0.039 ± 0.01^a^	0.029 ± 0.00^b^
C18:2n6c	2.58 ± 0.43	2.69 ± 0.19	2.63 ± 0.37
C20:0	0.07 ± 0.01	0.06 ± 0.02	0.06 ± 0.01
C20:1	0.10 ± 0.02	0.10 ± 0.02	0.09 ± 0.00
C20:2	0.11 ± 0.02	0.10 ± 0.03	0.08 ± 0.01
C20:3n6	0.08 ± 0.03	0.09 ± 0.02	0.10 ± 0.02
C20:4n6	0.54 ± 0.25	0.67 ± 0.22	0.79 ± 0.22
C24:0	0.05 ± 0.02^b^	0.08 ± 0.03^ab^	0.09 ± 0.02^a^

*Data are presented as means ± SE. Values in the same row without a common superscript letter are different (P < 0.05). Control group, n = 12, 11, and 8; sow betaine group, n = 6, 7, and 6; sow-offspring betaine group, n = 7, 7, and 6; at 65-, 95-, and 125-day-old, respectively*.

**Table 4:** Effect of dietary betaine addition on medium- and long-chain fatty acid contents in *biceps femoris* muscle of Bama mini-pigs (fresh weight basis; %).

**Items**	**Control group**	**Sow betaine group**	**Sow-offspring betaine group**
**65-day-old**			
Intramuscular fat	1.16 ± 0.07	1.24 ± 0.18	1.54 ± 0.26
C12:0	0.05 ± 0.01^a^	0.04 ± 0.01^b^	0.03 ± 0.01^b^
C14:0	0.36 ± 0.06	0.33 ± 0.04	0.32 ± 0.04
C15:0	0.02 ± 0.02	0.03 ± 0.02	0.02 ± 0.01
C16:0	6.71 ± 0.48	6.50 ± 0.30	6.68 ± 0.38
C16:1	1.03 ± 0.14	0.96 ± 0.10	1.07 ± 0.28
C17:0	0.11 ± 0.04	0.11 ± 0.03	0.10 ± 0.02
C18:0	3.69 ± 0.28	3.66 ± 0.23	3.64 ± 0.41
C18:1n9c	7.76 ± 0.66	7.49 ± 1.12	8.06 ± 0.91
C18:1n9t	0.04 ± 0.01	0.04 ± 0.01	0.03 ± 0.02
C18:2n6c	4.82 ± 0.50	4.64 ± 0.37	4.50 ± 0.70
C18:3n3	0.15 ± 0.01	0.14 ± 0.02	0.14 ± 0.01
C18:3n6	0.01 ± 0.02^b^	0.02 ± 0.02^ab^	0.03 ± 0.01^a^
C20:0	0.04 ± 0.01	0.04 ± 0.02	0.04 ± 0.01
C20:1	0.18 ± 0.04	0.16 ± 0.04	0.18 ± 0.03
C20:2	0.15 ± 0.02	0.13 ± 0.02	0.14 ± 0.02
C20:3n6	0.14 ± 0.02	0.13 ± 0.03	0.12 ± 0.02
C20:4n6	1.41 ± 0.38	1.32 ± 0.39	1.25 ± 0.36
C22:6n3	0.07 ± 0.02	0.06 ± 0.03	0.05 ± 0.01
C23:0	0.05 ± 0.02	0.04 ± 0.01	0.04 ± 0.02
C24:0	0.10 ± 0.04^a^	0.08 ± 0.02^ab^	0.06 ± 0.03^b^
**95-day-old**			
Intramuscular fat	1.08 ± 0.20	1.02 ± 0.17	1.28 ± 0.18
C14:0	0.37 ± 0.05	0.33 ± 0.04	0.39 ± 0.06
C15:0	0.02 ± 0.01	0.03 ± 0.01	0.02 ± 0.00
C16:0	6.75 ± 0.40	6.57 ± 0.37	6.97 ± 0.41
C16:1	0.76 ± 0.10	0.75 ± 0.13	0.80 ± 0.15
C17:0	0.08 ± 0.02	0.09 ± 0.02	0.07 ± 0.01
C18:0	4.18 ± 0.45	4.10 ± 0.15	4.08 ± 0.54
C18:1n9c	7.86 ± 0.97	7.71 ± 1.17	8.41 ± 0.73
C18:1n9t	0.03 ± 0.01	0.03 ± 0.01	0.03 ± 0.01
C18:2n6c	4.26 ± 0.74	4.08 ± 0.79	3.55 ± 0.53
C18:3n3	0.11 ± 0.01	0.11 ± 0.02	0.10 ± 0.01
C20:0	0.04 ± 0.02	0.04 ± 0.00	0.05 ± 0.01
C20:1	0.22 ± 0.06	0.19 ± 0.04	0.25 ± 0.04
C20:2	0.14 ± 0.03	0.13 ± 0.02	0.13 ± 0.02
C20:3n6	0.16 ± 0.07	0.14 ± 0.04	0.12 ± 0.03
C20:4n6	1.59 ± 0.80	1.38 ± 0.36	1.10 ± 0.17
C22:6n3	0.02 ± 0.03	0.04 ± 0.02	0.01 ± 0.02
C24:0	0.09 ± 0.03	0.08 ± 0.02	0.07 ± 0.02
**125-day-old**			
Intramuscular fat	1.87 ± 0.17	1.88 ± 0.10	1.92 ± 0.23
C14:0	0.41 ± 0.06	0.39 ± 0.05	0.37 ± 0.20
C16:0	7.50 ± 1.06	7.21 ± 0.42	5.17 ± 4.08
C16:1	0.85 ± 0.12	0.82 ± 0.14	0.84 ± 0.07
C17:0	0.09 ± 0.03	0.10 ± 0.05	0.08 ± 0.03
C18:0	4.31 ± 0.67	4.17 ± 0.71	3.57 ± 1.81
C18:1n9c	9.55 ± 1.22	9.76 ± 0.60	10.36 ± 1.04
C18:1n9t	0.04 ± 0.01	0.03 ± 0.01	0.03 ± 0.01
C18:2n6c	3.17 ± 0.63	2.86 ± 0.24	3.11 ± 0.63
C20:0	0.05 ± 0.01^a^	0.05 ± 0.01^b^	0.06 ± 0.01^a^
C20:1	0.10 ± 0.02	0.09 ± 0.01	0.14 ± 0.08
C20:2	0.10 ± 0.02	0.10 ± 0.02	0.11 ± 0.02
C20:3n6	0.11 ± 0.03	0.10 ± 0.02	0.11 ± 0.02
C20:4n6	0.99 ± 0.34^a^	0.15 ± 0.36^b^	0.39 ± 0.61^b^
C22:6n3	0.02 ± 0.02	0.02 ± 0.02	0.03 ± 0.08
C24:0	0.09 ± 0.03	0.08 ± 0.01	0.05 ± 0.04

*Data are presented as means ± SE. Values in the same row without a common superscript letter are different (P < 0.05). Control group, n = 12, 11, and 8; sow betaine group, n = 6, 7, and 6; sow-offspring betaine group, n = 7, 7, and 6; at 65-, 95-, and 125-day-old, respectively*.

**Table 5:** Effect of dietary betaine addition on medium- and long-chain fatty acid contents in *psoas major* muscle of Bama mini-pigs (fresh weight basis; %).

**Items**	**Control group**	**Sow betaine group**	**Sow-offspring betaine group**
**65-day-old**			
Intramuscular fat	1.40 ± 0.16	1.62 ± 0.10	1.13 ± 0.19
C12:0	0.04 ± 0.01	0.04 ± 0.01	0.04 ± 0.01
C14:0	0.40 ± 0.12	0.40 ± 0.12	0.30 ± 0.06
C15:0	0.03 ± 0.02	0.03 ± 0.02	0.02 ± 0.02
C16:0	7.50 ± 0.75	7.23 ± 0.89	6.63 ± 0.54
C16:1	0.93 ± 0.17	0.91 ± 0.18	0.77 ± 0.21
C17:0	0.13 ± 0.06	0.13 ± 0.04	0.11 ± 0.02
C18:0	4.39 ± 0.44	4.18 ± 0.42	4.09 ± 0.33
C18:1n9c	7.22 ± 1.08	7.20 ± 1.17	6.06 ± 0.54
C18:1n9t	0.04 ± 0.01^a^	0.02 ± 0.01^b^	0.02 ± 0.02^b^
C18:2n6c	5.24 ± 1.11	4.80 ± 0.89	5.54 ± 0.64
C18:3n3	0.15 ± 0.01	0.16 ± 0.02	0.14 ± 0.01
C18:3n6	0.03 ± 0.02	0.01 ± 0.01	0.02 ± 0.02
C20:0	0.05 ± 0.02	0.05 ± 0.01	0.04 ± 0.02
C20:1	0.18 ± 0.05	0.17 ± 0.04	0.14 ± 0.03
C20:2	0.15 ± 0.02	0.14 ± 0.01	0.13 ± 0.03
C20:3n6	0.14 ± 0.05	0.12 ± 0.06	0.14 ± 0.02
C20:4n6	1.38 ± 0.75	1.09 ± 0.80	1.62 ± 0.40
C22:6n3	0.05 ± 0.03	0.04 ± 0.04	0.07 ± 0.02
C23:0	0.07 ± 0.04	0.04 ± 0.03	0.07 ± 0.02
C24:0	0.08 ± 0.04	0.08 ± 0.05	0.10 ± 0.02
**95-day-old**			
Intramuscular fat	1.17 ± 0.12	1.15 ± 0.13	1.32 ± 0.18
C14:0	0.32 ± 0.05	0.31 ± 0.05	0.35 ± 0.05
C15:0	0.027 ± 0.01	0.032 ± 0.01	0.020 ± 0.01
C16:0	6.58 ± 0.32	6.81 ± 0.48	6.76 ± 0.48
C16:1	0.52 ± 0.06	0.58 ± 0.12	0.56 ± 0.09
C17:0	0.09 ± 0.03	0.11 ± 0.02	0.08 ± 0.01
C18:0	4.52 ± 0.52	4.53 ± 0.30	4.42 ± 0.51
C18:1n9c	6.17 ± 0.39	6.47 ± 0.72	6.91 ± 1.05
C18:1n9t	0.03 ± 0.01	0.03 ± 0.01	0.03 ± 0.01
C18:2n6c	5.13 ± 0.77^a^	5.00 ± 0.69^a^	4.11 ± 0.62^b^
C20:0	0.04 ± 0.02	0.05 ± 0.01	0.05 ± 0.01
C20:1	0.18 ± 0.06^a^	0.12 ± 0.01^b^	0.10 ± 0.01^b^
C20:2	0.15 ± 0.04	0.13 ± 0.02	0.13 ± 0.02
C20:3n6	0.17 ± 0.07	0.16 ± 0.04	0.13 ± 0.05
C20:4n6	1.77 ± 0.82	1.49 ± 0.20	1.17 ± 0.30
C22:6n3	0.03 ± 0.03	0.03 ± 0.02	0.01 ± 0.02
C24:0	0.09 ± 0.03	0.10 ± 0.01	0.09 ± 0.02
**125-day-old**			
Intramuscular fat	1.64 ± 0.13^a^	1.23 ± 0.04^b^	1.55 ± 0.08^a^
C14:0	0.38 ± 0.17	0.39 ± 0.10	0.42 ± 0.11
C15:1	2.65 ± 0.20	2.53 ± 0.22	2.70 ± 0.41
C16:1	0.67 ± 0.11	0.61 ± 0.11	0.63 ± 0.11
C17:0	0.12 ± 0.05	0.13 ± 0.07	0.11 ± 0.04
C18:0	5.05 ± 0.35	5.05 ± 0.65	4.09 ± 2.06
C18:1n9c	8.73 ± 0.69	8.54 ± 0.64	8.87 ± 1.06
C18:1n9t	0.03 ± 0.02	0.03 ± 0.02	0.03 ± 0.01
C18:2n6c	4.93 ± 0.58	4.95 ± 0.18	4.90 ± 0.63
C18:3n3	0.13 ± 0.02	0.12 ± 0.01	0.12 ± 0.01
C18:3n6	0.02 ± 0.02	0.03 ± 0.03	0.03 ± 0.02
C20:0	0.06 ± 0.01	0.05 ± 0.01	0.06 ± 0.01
C20:1	0.26 ± 0.06	0.25 ± 0.08	0.27 ± 0.10
C20:2	0.13 ± 0.02	0.12 ± 0.02	0.12 ± 0.02
C20:3n6	0.15 ± 0.03	0.16 ± 0.03	0.16 ± 0.02
C20:4n6	1.33 ± 0.33	1.43 ± 0.19	1.28 ± 0.21
C24:0	0.11 ± 0.06	0.15 ± 0.04	0.14 ± 0.03

*Data are presented as means ± SE. Values in the same row without a common superscript letter are different (P < 0.05). Control group, n = 12, 11, and 8; sow betaine group, n = 6, 7, and 6; sow-offspring betaine group, n = 7, 7, and 6; at 65-, 95-, and 125-day-old, respectively*.

### Medium- and Long-Chain Fatty Acid Contents in Muscles

The MCFA and LCFA contents in the LD muscle are presented in Table 3. Compared with the control group, betaine addition in sow-offspring diets increased (*P* < 0.05) the content of C24:0 and decreased (*P* < 0.05) the content of C18:1n9t in the 125-day-old pigs. Moreover, betaine addition in sow and sow-offspring diets increased (*P* < 0.05) the content of C12:0 in the 65-day-old pigs, while decreased (*P* < 0.05) the content of C12:0 in the 95-day-old pigs compared with the pigs in the control group. Compared with the sow betaine group, betaine addition in sow-offspring diets decreased (*P* < 0.05) the content of C18:3n6 in the 65-day-old pigs.

The MCFA and LCFA contents in the BF muscle are presented in Table 4. Compared with the control group, betaine addition in sow-offspring diets increased (*P* < 0.05) the content of C18:3n6 and decreased (*P* < 0.05) the content of C24:0 in the 65-day-old pigs, while betaine addition in sow diets decreased (*P* < 0.05) the content of C20:0 in the 125-day-old pigs. In addition, betaine addition in sow and sow-offspring diets decreased (*P* < 0.05) the contents of C12:0 in the 65-day-old pigs and C20:4n6 in the 125-day-old pigs, when compared with the control group.

The MCFA and LCFA contents in the PM muscle are presented in Table 5. Compared with the control group, betaine addition in sow and sow-offspring diets decreased (*P* < 0.05) the contents of C18:1n9t in the 65-day-old pigs and C20:1 in the 95-day-old pigs, while betaine addition in sow-offspring diets decreased (*P* < 0.05) the content of C18:2n6c in the 95-day-old pigs.

### The Expression Levels of Genes Related to Fatty Acid Metabolism in Muscles

**Figure 1:**
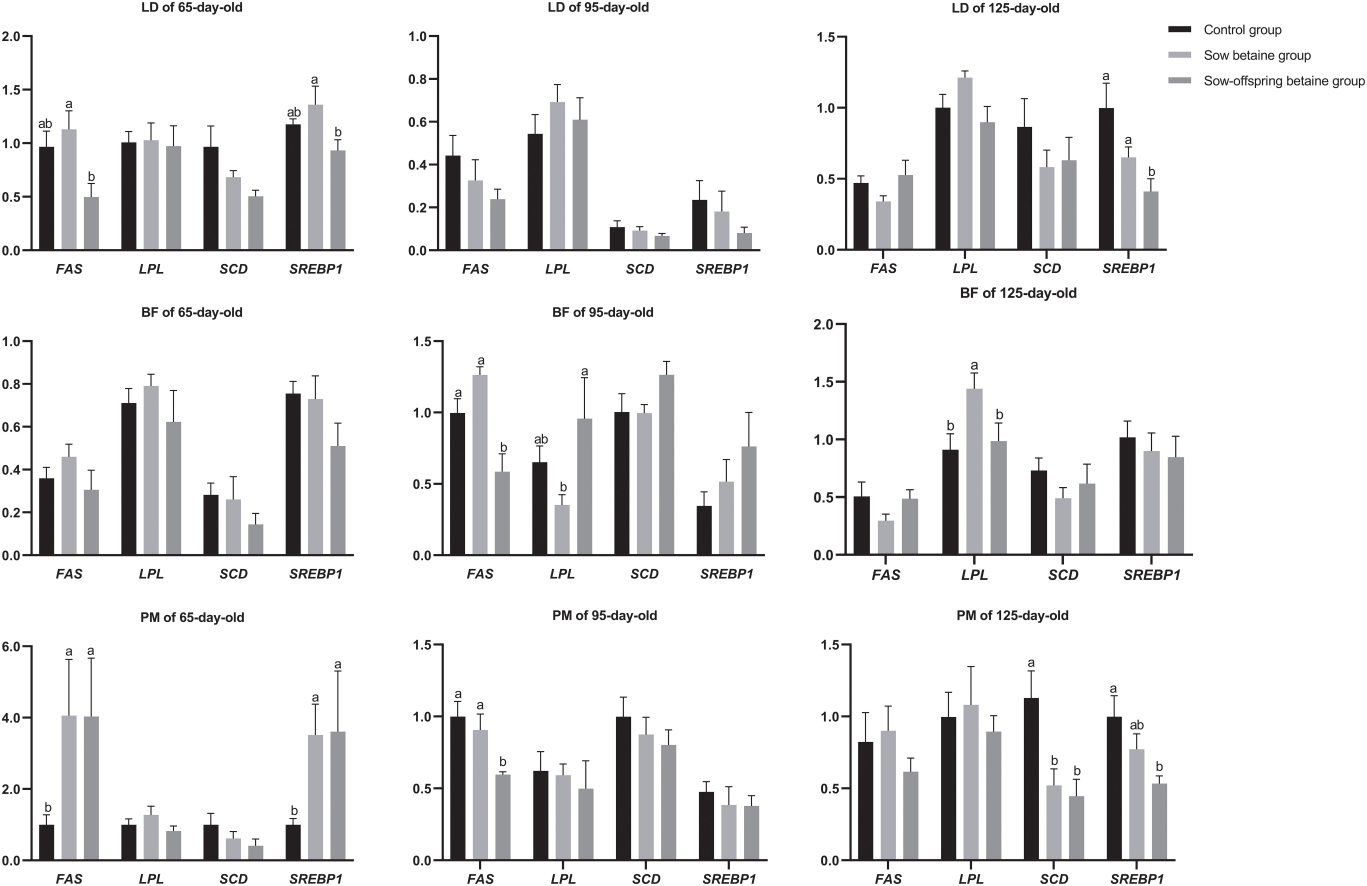
Effect of dietary betaine addition on the expression levels of genes related to lipid metabolism in muscles of Bama mini-pigs. Results are presented as the means ± SE. Bars marked with different lowercase letters are significantly different at *P* < 0.05. Control group, *n* = 12, 11, and 8; sow betaine group, *n* = 6, 7, and 6; sow-offspring betaine group, *n* = 7, 7, and 6; at 65-, 95-, and 125-day-old, respectively. *FAS*, fatty acid synthase; *LPL*, lipoprotein lipase; *SCD*, stearoyl coenzyme A desaturase; *SREBP1*, sterol regulatory element binding protein 1. BF, *biceps femoris* muscle; LD, *longissimus dorsi* muscle; PM, *psoas major* muscle.

The expression levels of genes related to fatty acid metabolism in the LD, BF, and PM muscles of pigs are shown in Figure 1. In the LD muscle, dietary betaine addition in sow-offspring diets down-regulated (*P* < 0.05) the mRNA expression levels of *FAS* and sterol regulatory element-binding protein 1 (*SREBP1*) in the 65-day-old pigs compared with the sow betaine group, as well as the mRNA expression level of *SREBP1* in the 125-day-old pigs compared with the pigs in the control and sow betaine groups. However, dietary betaine addition in sow and sow-offspring diets did not affect (*P* > 0.05) the gene expression levels of the LD muscle at 95-day-old pigs. In the BF muscle, dietary betaine addition in sow-offspring diets down-regulated (*P* < 0.05) the mRNA expression level of *FAS* compared with the pigs in the control and sow betaine groups, while up-regulated (*P* < 0.05) the mRNA expression level of *LPL* compared with the sow betaine group in the 95-day-old pigs. In addition, dietary betaine addition in sow diets up-regulated (*P* < 0.05) the mRNA expression level of *LPL* in the 125-day-old pigs compared with the control and sow-offspring betaine groups. Moreover, dietary betaine addition had no impacts (*P* > 0.05) on the mRNA gene expression levels of the BF muscle at 65-day-old pigs. In the PM muscle, compared with the control group, dietary betaine addition in sow and sow-offspring diets up-regulated (*P* < 0.05) the mRNA expression levels of the *FAS* and *SREBP1* in the 65-day-old pigs, whereas down-regulated (*P* < 0.05) the mRNA expression level of *SCD* in the 125-day-old pigs. In addition, dietary betaine addition in sow-offspring diets down-regulated (*P* < 0.05) the mRNA expression level of *SREBP1* in the 125-day-old pigs compared with the pigs in the control group. Furthermore, dietary betaine addition in sow-offspring diets down-regulated (*P* < 0.05) the mRNA expression level of *FAS* in the 95-day-old pigs compared with the control and sow betaine groups.

## Discussion

As an osmotic regulator, dietary betaine has been widely studied as a methyl donor on protein synthesis and nitrogen metabolism. Moreover, there are lots of creditability evidence showing that dietary betaine can influence growth performance and meat quality through its different functional properties. Previous studies have found that dietary betaine addition during the growing and finishing stages improved the growth performance and meat quality of animals (21, 22). In addition, maternal nutrition also influences the offspring's growth later in life (23). Therefore, the present study evaluated the effects of betaine addition in sow and sow-offspring diets on the growth performance and meat quality-associated parameters of pigs at different stages. The results showed that the betaine addition in sow and sow-offspring diets influenced the growth performance, plasma hormone, and lipid metabolism of Bama mini-pigs.

Betaine is widely used in animal production because of its positive effects on animal performance. In the present study, betaine addition in sow-offspring diets increased the BW of 125-day-old pigs, as well as the ADG and ADFI at 35–65 and 65–95 day-old. Therefore, the results indicate that betaine addition in sow-offspring diets can improve the growth of the piglets by enhancing the feed intake at an earlier age. Huang et al. (24) also found that dietary addition of 1.25 kg/t betaine for 42 days could increase the ADG of finishing pigs by 5.50%, but not the ADFI and F:G. In a sub-optimal methionine diet (15 g Met/kg CP), dietary betaine addition (0.80 kg/t) could improve the BW gain of broilers at 21-day-old, as well as the breast yield and F:G of broilers at 42-day-old (25). Furthermore, juvenile freshwater prawn *Macrobrachium rosenbergii* fed with 5 g/kg glycine betaine presented higher BW, food intake, and F:G (26). Therefore, these findings suggested that dietary betaine addition might increase the protein synthesis and decomposition through its methyl donor properties, thus increasing the growth and development of pigs.

Plasma hormone levels can reflect the physiological and health status of pigs. Growth hormone can regulate the growth and development of animals directly or indirectly by stimulating IGF-1 (27). Huang et al. (28) reported that dietary betaine addition could stimulate the release of GH and promote the growth of finishing pigs. However, the present study showed that betaine addition in sow and sow-offspring diets did not affect the plasma GH level, which might be related to the measured time of the plasma GH level. Kraetzl et al. (29) reported that the GH secretion is pulsatile, and its level is related to the measured time in pigs, that GH levels were higher during the night (20:00–08:00) than during the day (08:00–20:00). Therefore, the inconsistencies of these results might be related to the measuring time, as the present study only measured the GH level during the daytime. Gastrin and growth hormone-releasing factors could jointly promote GH secretion, regulate protein and sugar metabolism, and thus promote the growth of animals (30). As a type of gastrointestinal-derived hormone, PYY can inhibit feed intake, delay gastric emptying, increase the absorption of electrolytes in the ileum, and reduce the secretion of starch (31). The LEP is a protein hormone that can suppress the appetite and play a major role in regulating body fat, BW, and energy balance in animals (32). In the present study, betaine addition in sow diets decreased plasma PYY and LEP levels of 65-day-old pigs, while betaine addition in sow-offspring diets increased the Gas level of 95-day-old pigs, which are beneficial to promote the feed intake and growth of pigs.

The IMF mainly reflects muscle tenderness and flavor, and is a key factor to the formation of muscle taste. Existing studies showed that the IMF content between 2 and 3% is the best (33). In the present study, the IMF content was significantly decreased in the PM muscles of 125-day-old pigs but not in LD and BF muscles. However, Martins et al. (34) reported that 1.00 kg/t betaine addition in sow diets for 20 weeks could increase the IMF content in LD and BF muscles of Alentejano pigs. Huang et al. (35) found that 1.20 kg/t betaine addition in corn-soybean meal diets for 42 days increased the IMF content in LD muscle of finishing pigs. Therefore, these inconsistencies of IMF content might be related to the age of pigs. Moreover, the results suggested that dietary betaine mainly increased the IMF deposition in the earlier age of pigs, while could inhibit the IMF deposition in the later age.

The composition of fatty acid in the muscle plays an important role in the formation of pork flavor. High levels of unsaturated lipids may contribute to desirable flavors, like oxidized off-flavors (36). In the present study, dietary betaine addition tends to decrease the contents of PUFA, such as C18:1n9t, C18:3n6, C18:2n6c, and C20:4n6. These findings are in agreement with those of Madeira et al. (37), who reported that dietary betaine addition mainly decreases the percentage of monounsaturated fatty acid (including C16:1cis-9 and C18:1cis-11) proportions in the muscles of pigs. These findings might be resulted by the methyl donor properties of betaine. The principal physiologic role of betaine is as a methyl donor, which means transmethylation of betaine participates in the carnitine and phosphatidylcholine synthesis and fatty acid oxidation. Therefore, dietary betaine addition in sow diets may enhance the oxidation of PUFA and then reduce fatty acid and IMF contents (38, 39).

The *SREBP1* is a membrane-bound protein that can regulate the most enzymes involved in fatty acid synthesis, including acetyl-CoA carboxylase (*ACC*), *FAS*, and *SCD* (40, 41). The *LPL* is an important lipid regulatory enzyme involved in hydrolyzing triglycerides of plasma lipoproteins and supplying free fatty acids for storage in adipocytes or oxidation in other tissues (42). Moreover, *FAS* is a multifunctional protein, which main functional role is to catalyze the synthesis of palmitate from the acetyl-CoA and malonyl-CoA, in the presence of nicotinamide adenine dinucleotide phosphate (NADPH), into long-chain saturated fatty acid (43). In the present study, betaine addition in sow-offspring diets down-regulated the level of the *FAS* in the LD muscle of 65-day-old pigs, as well as in the BF and PM muscle of 95-day-old pigs, while dietary betaine addition in sow and sow-offspring diets up-regulated the level of *FAS* in the PM muscle of 65-day-old pigs. Moreover, betaine addition in sow diets up-regulated the level of *LPL* in the BF muscle of 125-day-old pigs while down-regulated in the BF muscle of 95-day-old pigs. Therefore, these findings suggest that dietary betaine can regulate fatty acid synthesis and fat deposition by enhancing lipogenesis and reducing lipolysis. Meanwhile, dietary betaine addition in sow and sow-offspring diets up-regulated the expression level of *SREBP1* in the PM muscle of 65-day-old pigs, while dietary betaine addition in sow-offspring diets down-regulated the *SREBP1* level in the LD and PM muscles of 125-day-old pigs. Thus, the present study showed that dietary betaine could improve the synthesis of fatty acids in the earlier age, while inhibiting the process in the later age, which is also consistent with the changes of the IMF content in the muscles of pigs.

## Conclusion

In summary, betaine addition in sow-offspring diets could improve the growth performance of piglets by increasing body weight, average daily gain, and average daily feed intake, decreasing the contents of several fatty acids, and inhibiting the fat deposition in muscles. Betaine addition in both sow and sow-offspring diets could influence the plasma hormones related to ingestion. Moreover, betaine addition in sow-offspring diets had more distinct effects than the addition in sow diets. These findings provide a reference for regulating lipid metabolism and promoting the growth of pigs in practical production by dietary betaine addition.

## Data Availability Statement

The original contributions presented in the study are included in the article/Supplementary Material, further inquiries can be directed to the corresponding author/s.

## Ethics Statement

The present study was conducted following Chinese guidelines for animal welfare and experimental protocols and approved by the Animal Care and Use Committee of the Institute of Subtropical Agriculture, Chinese Academy of Sciences, Changsha, China (ISA-2018-071).

## Author Contributions

YC, MS, QZ, and MA performed sampling and nutrient measurements, analyzed data, interpreted the data, and drafted the manuscript. MS and QG conducted animal feeding and sampling. XK contributed to experimental concepts and design, provided scientific direction, and together with MA finalized the manuscript. All authors read and approved the final manuscript.

## Funding

This work was supported by the National Natural Science Foundation of China (No. 31772613), Special Funds for Construction of Innovative Provinces in Hunan Province (2019RS3022), and Production and Research Talent Support Project of the CAS Wang Kuancheng Initiative Talent Program.

## Conflict of Interest

The authors declare that the research was conducted in the absence of any commercial or financial relationships that could be construed as a potential conflict of interest.

## Publisher's Note

All claims expressed in this article are solely those of the authors and do not necessarily represent those of their affiliated organizations, or those of the publisher, the editors and the reviewers. Any product that may be evaluated in this article, or claim that may be made by its manufacturer, is not guaranteed or endorsed by the publisher.
