# Supplementary material for: Impacts of Betaine Addition in Sow and Piglet's Diets on Growth Performance, Plasma Hormone, and Lipid Metabolism of Bama Mini-Pigs
Source: Front Nutr. 2021 Dec 23;8:779171. doi: 10.3389/fnut.2021.779171 (PMC8733558; doi:10.3389/fnut.2021.779171)
Supplement: Supplementary file 1 [file Data_Sheet_1.PDF]

Supplementary Material

Supplemental Table 1. Composition and nutrient levels of the basal diets for sows (air-dry basis; %)

Items	Pregnant sows' diet	Lactating sows' diet
Ingredients		
Corn	37.50	66.00
Soybean meal	9.50	25.00
Wheat bran	14.00	5.00
Barley	25.00	0.00
Soybean hull	10.00	0.00
Pregnant sows' premix[1)]	4.00	0.00
Lactating sows' premix[2)]	0.00	4.00
Total	100.00	100.00
Nutrient levels[3)]		
Digestive Energy (MJ/Kg)	12.55	13.87
Crude Protein	12.82	16.30
Lys	0.48	0.75
Met+Cys	0.43	0.51
Thr	0.37	0.53
Trp	0.13	0.17
Ca	0.62	0.65
Total P	0.47	0.50

[1)]Pregnant sows' premix provided the following per kg of the diet: Ca 2.32 g, Na 6.75 g, Cu 20.48 mg, Fe 118.60 mg, Zn 87.15 mg, Mn 32.54 mg, Mg 0.10 g, I 39.00 mg, Se 16.44 mg, Co 7.26 mg, VA 10 000 IU, VE 20.00 mg, VK_3 2.40 mg, VB_1 1.60 mg, VB_2 6.00 mg, VB_6 1.60 mg, VB_{12} 0.024 mg, folic acid 1.20 mg, nicotinamide 20.00 mg, pantothenic acid 12.00 mg, biotin 0.12 mg, ferrous glycinate 100.00 mg, choline chloride 1.00 g, phytase 200.00 mg, flavoring agent 80.00 mg, and limestone 12.00 g.

[2)] Lactating sows' premix provided the following per kg of the diet: Ca 2.32 g, Na 6.75 g, Cu 20.48 mg, Fe 118.60 mg, Zn 87.15 mg, Mn 32.54 mg, I 39.00 mg, Se 16.44 mg, Co 7.26 mg, VA 10 000 IU, VD_3 1 800 IU, VE 20.00 mg, VK_3 2.40 mg, VB_1 1.60 mg, VB_2 6.00 mg, VB_6 1.60 mg, VB_{12} 0.024 mg, folic acid 1.20 mg, nicotinamide 20.00 mg, pantothenic acid 12.00 mg, biotin 0.12 mg, ferrous glycinate 100.00 mg, choline chloride 1.00 g, phytase 200.00 mg, flavoring agent 80.00 mg, and limestone 12.00 g.

[3)] Nutrient levels were calculated values.

Supplemental Table 2. Composition and nutrient levels of basal diets for weaned pigs (air-dry basis; %)

Items	Pre-nursery diet (35-95 day-old)	Post nursery diet (96-125 day-old)
Ingredients		
Corn	54.92	58.00
Soybean meal	22.00	18.35
Wheat bran	10.13	11.35
Rice bran	8.95	8.30
Premix[1)]	4.00	4.00
Total	100.00	100.00
Nutrient levels[2)]		
Digestive Energy (MJ/kg)	13.50	13.42
Crude Protein	16.13	14.90
Lys	1.40	1.30
Met + Cys	0.69	0.66
Thr	0.78	0.74
Ca	0.45	0.44
Total P	0.49	0.49

[1)] Premix provided the following per kg of diets: enzyme preparation 1.2 g, VA 26 000 IU, VD_3 10 000 IU, VE 70 IU, VK_3 10 mg, VB_1 10 mg, VB_2 25 mg, VB_6 10 mg, VB_{12} 0.075 mg, biotin 0.4 mg, folic acid 5 mg, nicotinamide 100 mg, pantothenic 50 mg, choline 1 600 mg, flavoring agent 500 mg, edulcorant 300 mg, acidulating agent 5 g, Cu 230 mg, Mn 97 mg, Zn 218 mg, Fe 165 mg, I 0.3 mg, Se 0.3 mg, Co 0.4 mg, glucose 2.1 g, antioxidants 0.4 g, anti-mildew agent 1.0 g, Ca 3.42 g, and P 1.155 g.

[2)] Nutrient levels were calculated values.

Supplemental Table 3. Primer sequences for the target genes

Target Genes	GeneBank No.		Primer Sequences (5′-3′)
β-Actin	XM_021086047.1	F:	GGCACCACACCTTCTACAACGAG
		R:	TCATCTTCTCACGGTTGGCTTTGG
FAS	NM_001099930.1	F:	CCACTCCAAGCAGGCGAACAC
		R:	CACGAAGGGAAGCAGGGTTGAT
LPL	XM_021072174.1	F:	ACACAGTTGAGGACACTTGCCATC
		R:	TCCTGTCACCGTCCAGCCATG
SCD	NM_213781.1	F:	CCACAACTACCACCACACCTTTCC
		R:	AGCCATGCAGTCGATGAAGAACG
SREBP-1	XM_021066226.1	F:	TGCTGGCGGAGGTCTATGTGG
		R:	CAGGAAGAAGCGGGTCAGAAAGTG

FAS, fatty acid synthase, *LPL*, lipoprotein lipase, *SCD*, stearoyl coenzyme A desaturase *SREBP1*, sterol regulatory element binding protein 1.
